# A traditional botanical formula attenuates acute pharyngitis in mice through anti-inflammatory and anti-staphylococcal activities

**DOI:** 10.3389/fmicb.2026.1841770

**Published:** 2026-05-22

**Authors:** Xinyu Zhang, Wenyuan Li, Qinghua Xie, Yifei Zhang, Zerun Wang, Wan Wang, Ziqi Zhang, Rui Han

**Affiliations:** 1 Shaanxi Key Laboratory of Brain Disorders & Institute of Basic and Translational Medicine, Xi’an Medical University, Xi’an, China; 2 School of Stomatology, Xi’an Medical University, Xi’an, China; 3 School of General Medicine, Xi’an Medical University, Xi’an, China; 4 School of Pharmacy, Xi’an Medical University, Xi’an, China; 5 School of Clinical Medicine, Shandong Second Medical University, Weifang, China

**Keywords:** acute pharyngitis, anti-inflammatory, anti-staphylococcal, botanical formula, network pharmacology, NF-κB, PI3K/AKT, *Staphylococcus aureus*

## Abstract

**Introduction:**

Acute pharyngitis is a common upper respiratory tract infectious disease worldwide. The increasing prevalence of multidrug‑resistant pathogens, particularly methicillin‑resistant *Staphylococcus aureus* (MRSA), greatly hinders clinical treatment and predisposes patients to recurrent and persistent inflammation. Consequently, there is an urgent need for safe and effective alternative therapeutic agents.

**Methods:**

This research focused on examining the protective effects of a traditional sore‑throat composite phytoextract (TSCP) formulated from edible medicinal natural ingredients, while also investigating its underlying mechanisms. An ammonia‑induced acute pharyngitis murine model was employed. Network pharmacology was used to screen potential bioactive components and key therapeutic targets, followed by the molecular biological validation. In vitro assays assessed the antibacterial and anti‑biofilm activities of TSCP.

**Results and discussion:**

TSCP dose‑dependently ameliorated abnormal behavioral manifestations, alleviated pharyngeal histopathological injury, and reduced inflammatory cell infiltration in the model mice. Network pharmacology identified 22 potential bioactive components and 174 key therapeutic targets, which were mainly enriched in the PI3K/AKT and NF‑κB signaling pathways. Subsequent molecular validation confirmed that TSCP modulated the PI3K/AKT/NF‑κB signaling axis and significantly downregulated the downstream inflammatory mediators iNOS, COX‑2, and IL‑6. Hematological results showed that TSCP decreased peripheral leukocyte, lymphocyte, and neutrophil levels, indicating a favorable systemic anti‑inflammatory effect. *In vitro* antimicrobial assays demonstrated that TSCP exhibited broad‑spectrum activity against *S. aureus*, MRSA, and *E. coli* with minimum inhibitory concentration (MIC) values of 32, 64, and 256 μg/mL, respectively; and the minimum bactericidal concentration (MBC) for each strain was twice its corresponding MIC. TSCP also concentration‑dependently inhibited bacterial adhesion, disrupted cell membrane integrity, and destroyed biofilm microstructure. Collectively, TSCP exerts prominent protective effects against acute pharyngitis through dual mechanisms: suppression of the inflammatory response via the PI3K/AKT/NF‑κB pathway, and direct broad‑spectrum antibacterial and anti‑biofilm activities *in vitro*. This study provides solid experimental evidence supporting the modern application of the compound herbal preparation TSCP, suggesting that TSCP is a promising candidate for the prevention and adjunctive treatment of drug‑resistant bacteria‑associated acute pharyngitis.

## Graphical abstract

**Figure:**
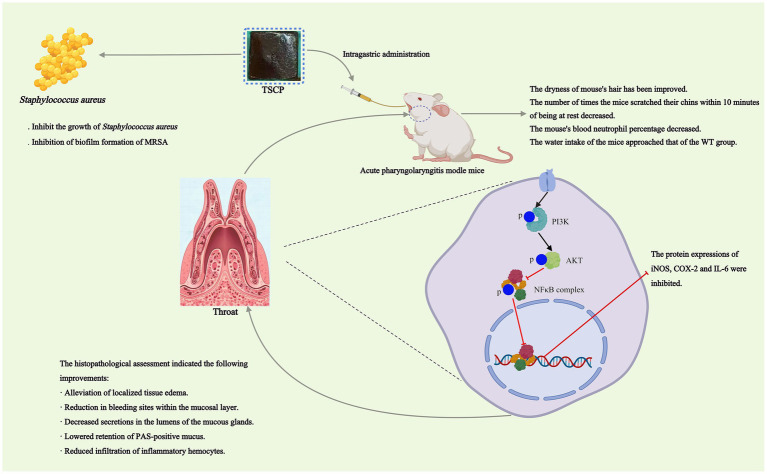

## 1 Introduction

Acute pharyngitis is a typical respiratory infectious disease that has become more widespread globally, with an increasing incidence (Okonogi et al., 2023). Although often of viral origin, bacterial pathogens play a significant role, with *Staphylococcus aureus* standing out as a clinically prominent causative agent. Recent surveillance data derived from clinical throat swabs indicate a detection rate of *S. aureus* as high as 57.3% in patients with acute pharyngitis. The pathogen exerts its virulence through mucosal adhesion and colonization, secretion of diverse toxins that compromise host cell integrity and subvert immune defenses, thereby inducing local inflammatory responses such as pharyngeal erythema and edema. These processes not only intensify patient discomfort but also substantially increase the risk of clinical deterioration.

Without timely and adequate treatment, frequent or recurrent episodes of acute pharyngitis may lead to laryngeal swelling. Glottic edema can cause dyspnea and, in severe cases, airway obstruction – a life-threatening condition. Deep neck space infections are also potential severe sequelae (Yin, 2019; Wu et al., 2021; Li, 2014). Current therapeutic strategies focus on controlling infection, reducing inflammation, relieving smooth muscle spasm, and improving mucosal edema, with the overall goal of alleviating airway inflammation and symptoms (Han and Fan, 2010; Zhang et al., 2020; Wei, 2019). However, traditional antibiotics have limited efficacy against drug-resistant bacteria such as methicillin-resistant *Staphylococcus aureus* (MRSA), underscoring the urgent need for novel antimicrobial agents (Xu et al., 2026; Dai et al., 2024; Liu et al., 2024; Zhong et al., 2026). Recent studies have shown that rationally designed natural product scaffolds offer a promising strategy against MRSA. For example, compound 5 dL, derived from rutaecarpine with a pyridinium quaternary ammonium “tail,” achieves complete bacterial eradication within 6 h through membrane perturbation and inhibition of DNA replication, and demonstrates >97% cure rate in a murine skin abscess model (Dai et al., 2024). Compound 9b, based on a harmane scaffold, interferes with bacterial metabolism via multi-target engagement, while compound H1 (a xanthotoxin derivative bearing a quaternary ammonium salt) induces precise membrane disruption. Both compounds display high potency, low cytotoxicity, and negligible resistance development even after 20 consecutive passages (Liu et al., 2024; Zhong et al., 2026). Collectively, these findings indicate that natural products or optimized natural products can effectively counteract MRSA resistance.

Drawing on the long history and theoretical basis of traditional Chinese medicine (TCM) in treating pharyngitis, such as “nourishing yin, moistening the lung, soothing the throat and removing toxicity” (Li et al., 2023; Xu et al., 2024; Zhou et al., 2023; Huang et al., 2024). Guided by the concept of “medicinal and edible homology” (Shi and Jiang, 2019), the present study developed a natural herbal composite named Traditional Sore-throat Composite Phyto-extract (TSCP). TSCP is a modified pear paste formula derived from the Collection of *Qing Palace Formulas*, combining Shaanxi Pucheng crisp pears with nine medicinal and edible materials, including *Ophiopogon japonicus*, *Fritillaria taipaiensis*, and *Mentha haplocalyx*. To systematically explore its mechanism of action along the “active ingredients–signaling pathways–acute pharyngitis” axis, we employed an integrated approach combining network pharmacology, molecular biology, and microbiology. Network pharmacology was used to screen bioactive components and predict targets and pathways. Animal experiments confirmed the therapeutic effect of TSCP in a murine model of acute pharyngitis, and molecular biology techniques further elucidated the underlying mechanisms. In addition, a series of *in vitro* microbiological assays were performed to evaluate the antibacterial potential of TSCP. Specifically, we determined the minimum inhibitory concentration (MIC) and minimum bactericidal concentration (MBC) of TSCP against three bacterial strains (*S. aureus* ATCC12600, MRSA ATCC43300, and *E. coli* CVCC216) using agar dilution and subculture methods. The results showed that TSCP exhibited bactericidal activity against all three strains, with MBC/MIC ratios of 2 for staphylococci and 4 for *E. coli*. Furthermore, crystal violet staining demonstrated that TSCP dose-dependently inhibited biofilm formation in all three strains. The anti-biofilm effect was further validated by SYTO9/propidium iodide (PI) live/dead staining and confocal laser scanning microscopy (CLSM), which revealed that TSCP disrupts the membrane integrity and three-dimensional architecture of MRSA biofilms. Collectively, these *in vitro* findings indicate that TSCP possesses broad-spectrum antibacterial activity against both Gram-positive and Gram-negative bacteria, as well as potent anti-biofilm activity against MRSA.

## 2 Materials and methods

### 2.1 Preparation of TSCP

Fresh crisp pears and fresh lotus roots were washed, crushed, and pressed to obtain the juices. Centrifugation and filtration were used to separate solids from liquids, and only the liquid part was kept. There 300 g of lotus root juice and 600 g of pear juice were weighed. Red dates (75 g), *Ophiopogon japonicus* (18.6 g), and *Fritillaria taipaiensis* (18.6 g) were added to the mixed juice, which was then boiled for 1 h and the herbal residue was removed. The extract was then sterilized by passing through a 0.22 μm PES membrane filter. Next, mint (*Mentha haplocalyx*, 18.6 g), persimmon sugar (100 g), and honey (10 g) were added to the filtrate. The mixture was concentrated under reduced pressure for less than an hour to obtain a viscous liquid or a solid after dissolution in some solvents. This crude extract was quickly frozen at −80 °C and then lyophilized in a freeze dryer for 48 h to obtain the dry powder. The freeze-dried TSCP powder was stored at −20 °C until it was needed.

Animal experiments were carried out in the laboratory, at which time the lyophilized TSCP powder needed to be accurately weighed, dissolved in sterile normal saline for oral administration to the treatment groups, while the control groups received an equal volume of sterile normal saline alone. This provided the working solutions for oral gavage at low (200 mg/kg/d), medium (400 mg/kg/d), and high (600 mg/kg/d) dose levels. A set volume of 100 μL was dispensed; this quantity was consistent across each group.

### 2.2 Animal housing and model establishment

All animals were kept in an SPF environment and had access to food and water freely; they were maintained on a 12-h light/dark cycle at a room temperature of 25 °C ± 1 °C and a relative humidity of 50%. To set up an acute pharyngitis model in mice, they were exposed to a 5% ammonia solution. Forty mice were randomly divided into five groups (eight per group).

The mice were anesthetized with 5% isoflurane inhalation, and 50 μL of a 5% ammonia solution was sprayed directly into the pharyngeal cavity using a micro-sprayer attached to a syringe. The procedure was performed gently to avoid mechanical injury. Starting from the first day of the model, the treatment group was given the TSCP solution by oral gavage for 12 days; the control group was administered an equal volume of saline.

All animal experimental procedures were strictly conducted in accordance with the Guide for the Care and Use of Laboratory Animals (Eighth Edition, ISBN-10: 0–309-15396-4) and were approved by the Animal Ethics Committee of Xi’an Medical University (Approval No. XYLS2026004).

(1) The Wild-type blank control group (WT group): saline stimulation + oral administration of saline (100 μL)

(2) The Model group (AP model group): 5% ammonia stimulation + oral administration of saline (100 μL)

(3) The Low Concentration Group for Acute Pharyngitis Treatment (AP-L group): 5% ammonia stimulation + oral administration of low concentration TSCP solution (200 mg/kg/d, 100 μL)

(4) The Medium Concentration Group for Acute Pharyngitis Treatment (AP-M group): 5% ammonia stimulation + oral administration of medium concentration TSCP solution (400 mg/kg/d, 100 μL)

(5) The High Concentration Group for Acute Pharyngitis Treatment (AP-H group): 5% ammonia stimulation + oral administration of high concentration TSCP solution (600 mg/kg/d, 100 μL)

### 2.3 Behavioral observation

From the day of the model, the scratching behavior of mice was recorded every other day from 9:00 to 11:00 a.m. in a soundproof observation room with constant temperature and humidity. Each group of mice was observed individually for 10 min, and the number of different scratching behaviors was recorded, excluding other behaviors that were not scratches, such as self-grooming and limb extension.

### 2.4 Physiological parameter measurement

Starting on the first day of modeling, body weight and blood glucose levels were measured every other day between 9:00 and 10:00 a.m. After modeling completion, the daily water intake was recorded at the same time for seven consecutive days.

### 2.5 Blood cell analysis

Mice (weighing 50–65 g) were deeply anesthetized with 5% (v/v) isoflurane in 100% oxygen delivered via a small animal anesthesia machine, with an oxygen flow rate of 0.5 L/min, until unresponsive to a firm toe pinch. Under this surgical plane of anesthesia, blood was collected via the abdominal aorta. Immediately after blood collection, while still under deep anesthesia, the mice were euthanized by cervical dislocation. Death was confirmed by respiratory arrest and cessation of heartbeat. Subsequently, pharyngeal tissues were harvested for further analysis.

After anesthesia, blood was drawn from the abdominal aorta and added to vacuum tubes containing EDTA-K₂ anticoagulant. The samples were placed in a refrigerator at 4 °C and tested after about 1 week. In accordance with the operating instructions of the hematology analyzer, samples were added to the instrument and quantitative determination of red blood cells (RBCs), hemoglobin (Hb), white blood cells (WBCs) and their subtypes, as well as platelets (PLTs), was performed. The raw data were collected and subsequently employed in the following stages of analysis.

### 2.6 HE staining, PAS staining, and immunofluorescence staining

After euthanasia, pharyngeal tissues were obtained and fixed in 4% paraformaldehyde (20 times the volume of the tissue). Decalcification, dehydration, paraffin embedding, and sectioning were performed, followed by sequential steps of HE staining, PAS staining, and immunofluorescence staining. The mucosal morphology and the mucus gland distribution were observed using a light microscope, the quantity of PAS-positive areas was determined, and immunofluorescence images were obtained and analyzed.

### 2.7 Caspase-3 and Caspase-9 activity assay

Pharyngeal tissue lysates and control samples were added directly to the assay plate. After sealing, the plate was incubated at 37 °C for 1–2 h. After washing, a biotinylated antibody was added and incubated for 1 h; then, after washing again, an enzyme conjugate was added and incubated in the dark for 30 min. Subsequently, the plate was washed. The TMB substrate was added and allowed to react in the dark until a clear color gradient appeared in the standard wells. A few drops of concentrated sulfuric acid were added to stop the reaction, and the optical density at 450 nm was read after 10 min.

### 2.8 Network pharmacology analysis

Based on the TCMSP database,^1^ the primary active components of the compounds formulation were selected according to the criteria that oral bioavailability (OB) ≥ 30% and drug likeness (DL) ≥ 0.18. Then, the SMILES structures of the selected active compounds were introduced into the SwissTargetPrediction and NetInfer platforms for predicting the action targets. The keyword “Acute Pharyngitis” was used to query the OMIM, GeneCards, and MalaCards databases for disease-related targets. The intersection of compound targets and disease targets was taken, and a protein–protein interaction network was built using the STRING database (confidence > 0.9). Key targets were imported into the DAVID 6.8 database for GO functional annotation and KEGG pathway enrichment analysis, and the results were visualized using a bioinformatics cloud platform. The “component-target-pathway-disease” network diagram was built with Cytoscape 3.9.1.

### 2.9 Western blotting

Throat tissue was homogenized in RIPA lysis buffer containing PMSF, and after centrifugation at 4 °C, the supernatant was collected. Protein content was determined using the BCA method. After denaturation, sample loading and SDS-PAGE electrophoresis, the proteins were transferred to a PVDF membrane, blocked with 5% non-fat milk for 1 hour, incubated with the primary antibody overnight at 4 °C, and then incubated with the secondary antibody at room temperature for 1 hour; finally, the signal was detected by ECL.

### 2.10 Determination of minimum inhibitory concentration (MIC) and minimum bactericidal concentration (MBC)

The MIC of TSCP against *Staphylococcus aureus* (*S. aureus*, ATCC12600), methicillin resistant *S. aureus* (MRSA, ATCC43300) and *Escherichia coli* (*E. coli*, CVCC216) were determined using the broth microdilution method according to CLSI guidelines. TSCP was serially two-fold diluted in Luria-Bertani (LB) to final concentrations of 0, 4, 8, 16, 32, 64, 128, 256, 512, 1,024 and 2048 μg/mL in 96 well microplates. Bacterial suspensions were adjusted to 10^7^ CFU/mL and added to each well. After incubation at 37 °C for 18–20 h, the MIC was defined as the lowest concentration that completely inhibited visible bacterial growth.

For MBC determination, 10 μL aliquots from each well showing no visible growth were spread onto Mueller Hinton agar plates without TSCP. After incubation at 37 °C for 24 h, the MBC was defined as the lowest concentration that killed ≥99.9% of the original inoculum (no colony formation). All tests were performed in triplicate on three independent occasions.

### 2.11 Live/dead bacterial staining by confocal microscopy

The bactericidal effect of TSCP was visualized using SYTO9/PI dual fluorescence staining. Bacterial suspensions (1 × 10^8^ CFU/mL) of the three strains were treated with different concentrations of TSCP for 3 h at 37 °C. After centrifugation, the pellets were stained with 2 μM SYTO9 and 4.5 μM propidium iodide (PI) for 60 min in the dark, followed by three washes with sterile water. Stained samples were observed under a confocal laser scanning microscope (Leica Microsystems, Germany). Live bacteria (SYTO9 stained) appeared green, while dead bacteria (PI stained) appeared red.

12. Anti biofilm activity assay by crystal violet staining.

The effect of TSCP on biofilm formation was assessed using the crystal violet method. Mature biofilms were prepared by separately adding 100 μL of *S. aureu*, *E. coli* and MRSA suspension (3.2 × 10^7^ CFU/mL) into 12 well plates containing 2 mL of tryptic soy broth (TSB) and incubated at 37 °C for 48 h. After biofilm maturation, the medium was removed, and the biofilms were treated with different concentrations of TSCP (0.5 × MIC, 1 × MIC, 2 × MIC) or PBS as control for 30 min. The biofilms were then gently washed three times with PBS, stained with 0.1% (w/v) crystal violet solution for 15 min, and washed again. The remaining stained biofilm was dissolved in 33% (v/v) acetic acid, and the absorbance was measured at 595 nm using a microplate reader (Thermo Fisher Scientific, USA). Finally, the biofilm was dissolved in a 33% acetic acid solution, and UV–vis absorbances of all samples at 595 nm were measured to assess the ability of biofilm formation. Each concentration was tested in triplicate and the experiment was repeated three times.

To evaluate the disruption of pre formed biofilms, mature MRSA biofilms prepared as above were incubated with different concentrations of TSCP (0.5 × MIC, 1 × MIC, 2 × MIC) for 3 h at 37 °C. After incubation, the biofilms were stained with SYTO9 (2 μM) for 30 min in the dark, washed gently, and then examined under a CLSM to assess biofilm integrity and thickness. 3D images were acquired to visualize structural changes.

### 2.12 Statistical analysis

Data were analyzed using SPSS 19.0 and expressed as mean ± SD. Normality was tested by the Shapiro–Wilk. One-way ANOVA with Tukey’s *post hoc* was applied for normally distributed multiple groups, and the Kruskal–Wallis with Dunn’s post hoc for non-normal data. Two-group comparisons used a *t*-test or the Mann–Whitney U. Significance was set at *p* < 0.05. Graphs were generated with GraphPad Prism 8.0.1.

## 3 Result

### 3.1 Physiological status of mice

**Figure 1:**
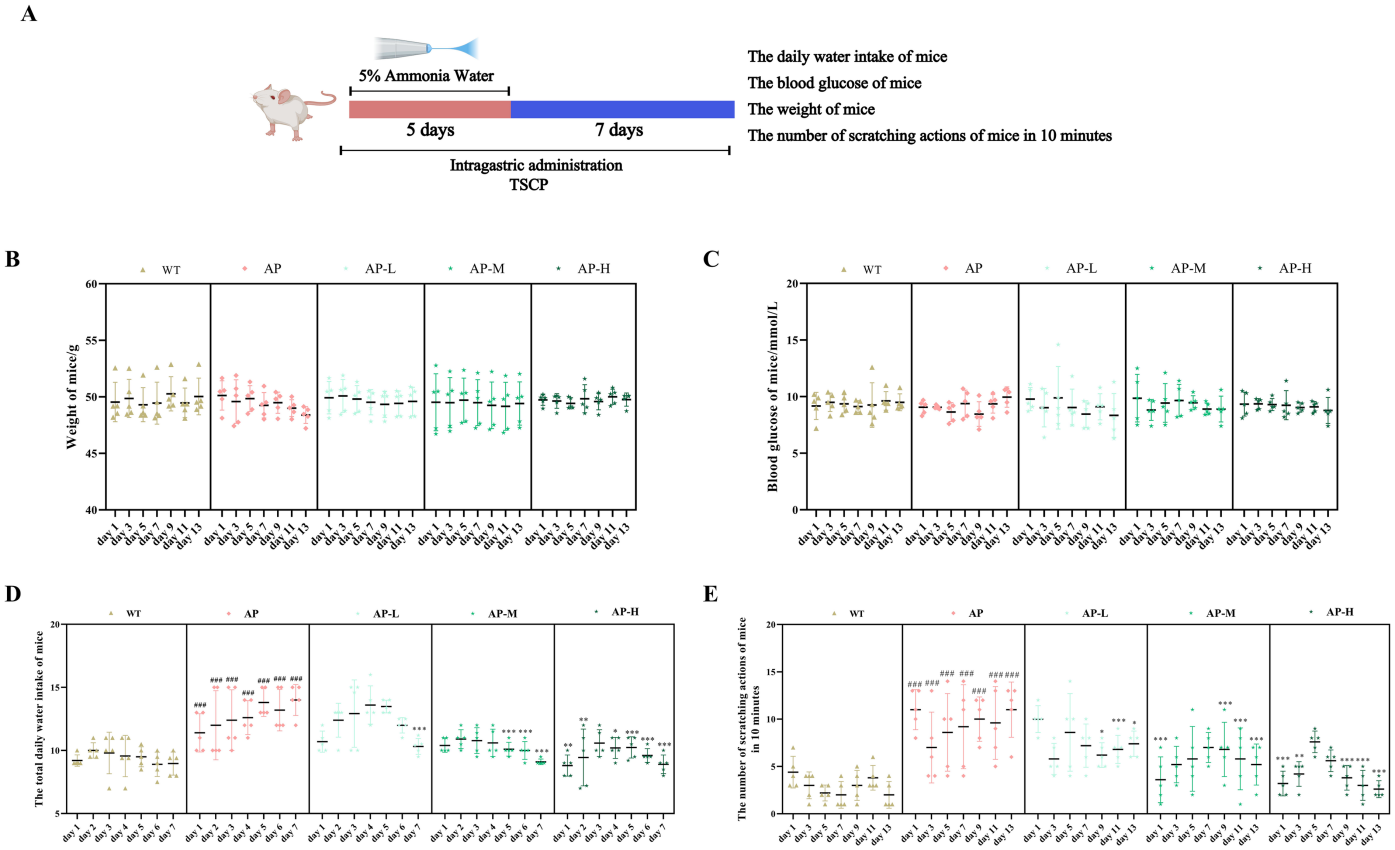
Effects of TSCP on physiological status and behavioral performance in mice with AP. **(A)** Establishment of the acute pharyngitis mouse model and measurement of physiological parameters. **(B)** The daily weight of mice. **(C)** The blood glucose of mice. **(D)** The total daily water intake of mice. **(E)** The number of scratching actions of mice in 10 min. Compared with the WT group, ^#^*p* < 0.05, ^##^*p* < 0.01, ^###^*p* < 0.001; compared with the AP model group ^*^p < 0.05, **p < 0.01, ***p < 0.001. *p* > 0.05 indicates no significant difference, and such comparisons are not marked.

Water intake, body weight, scratching frequency (within 10 min at rest), and blood glucose levels were measured in each group. As shown in Figure 1, compared with the WT group, mice in the AP model group exhibited rougher and duller fur, increased irritability during handling, and a significantly higher scratching frequency throughout the 13-day experimental period (### *p* < 0.001 on each testing day). The TSCP-treated groups showed dose-dependent behavioral improvements, with the high-dose group demonstrating the most pronounced effect (on day 13: AP-L = 7.4 ± 1.34, *p < 0.05; AP-M = 5.2 ± 2.05, ***p < 0.001; AP-H = 2.6 ± 0.89, ***p < 0.001 vs. the AP model group). No significant differences in body weight or blood glucose levels were observed among the experimental groups (*p* > 0.05). For water intake, TSCP treatment also reduced the elevated intake caused by pharyngitis in a dose-dependent manner. On day 7 after model establishment, all three doses significantly lowered water intake compared with the AP model group (AP-L = 10.32 ± 0.63 mL, ***p < 0.001; AP-M = 9.10 ± 0.22 mL, ***p < 0.001; AP-H = 8.90 ± 0.74 mL, ***p < 0.001 vs. the AP model group).

### 3.2 TSCP alleviated pathological manifestations in murine pharyngitis

**Figure 2:**
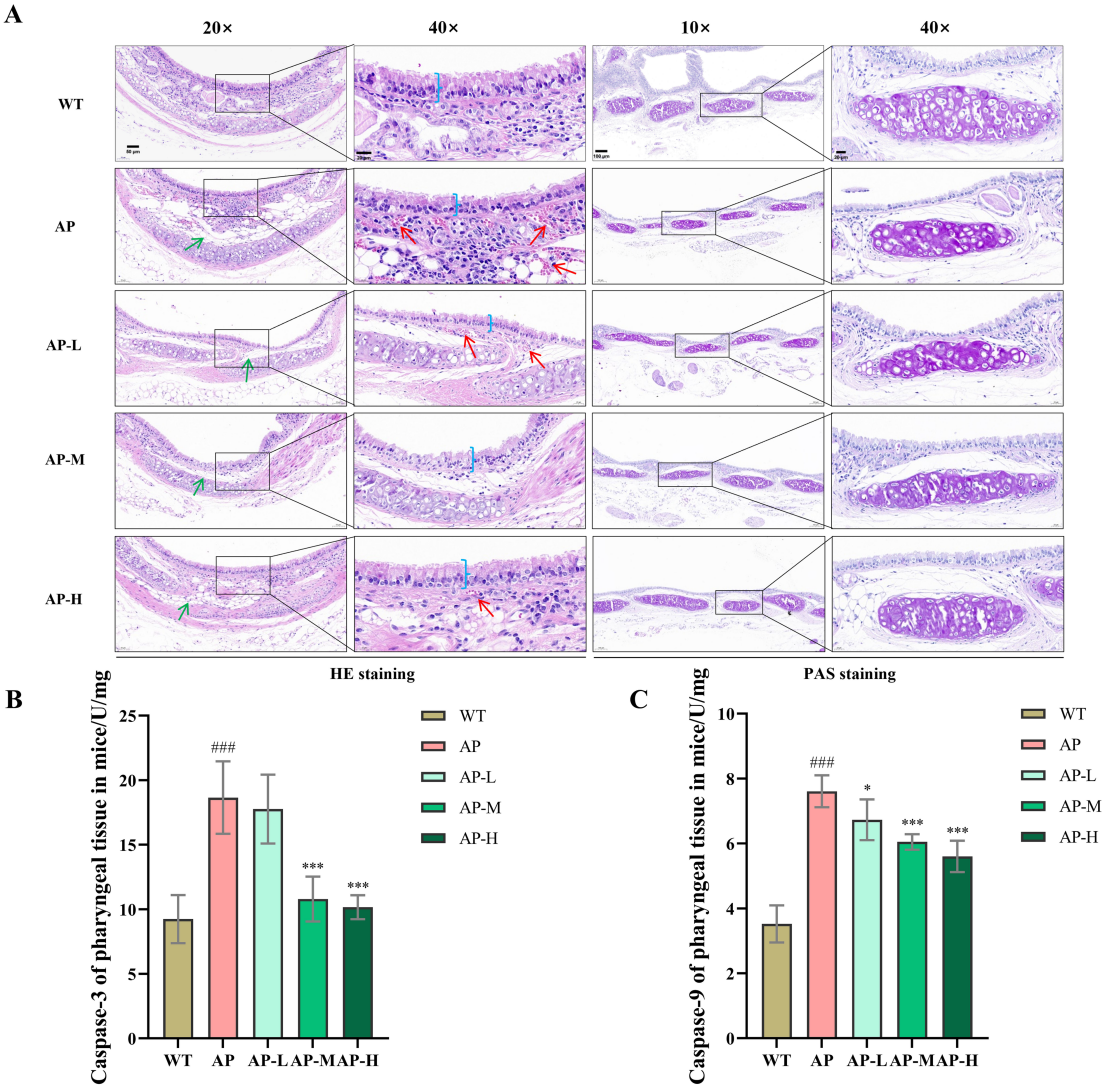
Histopathological improvement of acute pharyngitis by TSCP in mice. **(A)** Histopathology analysis of pharyngeal tissue. **(B)** Caspase-3 of pharyngeal tissue in mice. **(C)** Caspase-9 of pharyngeal tissues in mice. Compared with the WT group, ^#^*p* < 0.05, ^##^*p* < 0.01, ^###^*p* < 0.001; compared with the AP model group ^*^p < 0.05, **p < 0.01, ***p < 0.001. *p* > 0.05 indicates no significant difference, and such comparisons are not marked.

As shown in Figure 2A, HE and PAS staining showed that the WT group had a complete mucosal epithelium with regularly arranged cells, no significant inflammatory cell infiltration in the lamina propria, and normal secretion of mucus glands. In contrast, the AP model group had disorganised and partially exfoliated mucosal epithelial cells, extensive inflammatory cell infiltration in the lamina propria, marked tissue oedema, hyperactive secretion of mucous glands, and mucus retention. Compared with the AP model group, the treatment groups showed improved mucosal pathology, with more regularly arranged epithelium, reduced inflammatory cell infiltration, alleviated tissue edema, and decreased secretion of mucous glands as well as mucus retention. The pathological degree showed a dose–response relationship, and among them, the high-dose group achieved the best therapeutic effect on acute pharyngitis.

As shown in Figures 2B,C, the caspase-3 and caspase-9 expression levels in the pharyngeal tissue of the AP model group were significantly higher than those in the WT group (all *p* < 0.001). Compared with the AP model group, all treatment groups showed dose-dependent down-regulation of both proteins: for caspase-3, the low-dose group had no significant difference, while the medium- and high-dose groups were most inhibited (all *p* < 0.01); for caspase-9, a significant suppression effect was observed in the low-dose group (*p* < 0.05), and both the medium--dose group and the high-dose group also showed strong inhibition (both *p* < 0.01). Based on the above experimental results, it can be concluded that this formula is expected to inhibit apoptosis-related proteins caspase-3 and caspase-9 in a dose-dependent manner.

### 3.3 Screening of active ingredients and target prediction of TSCP

**Figure 3:**
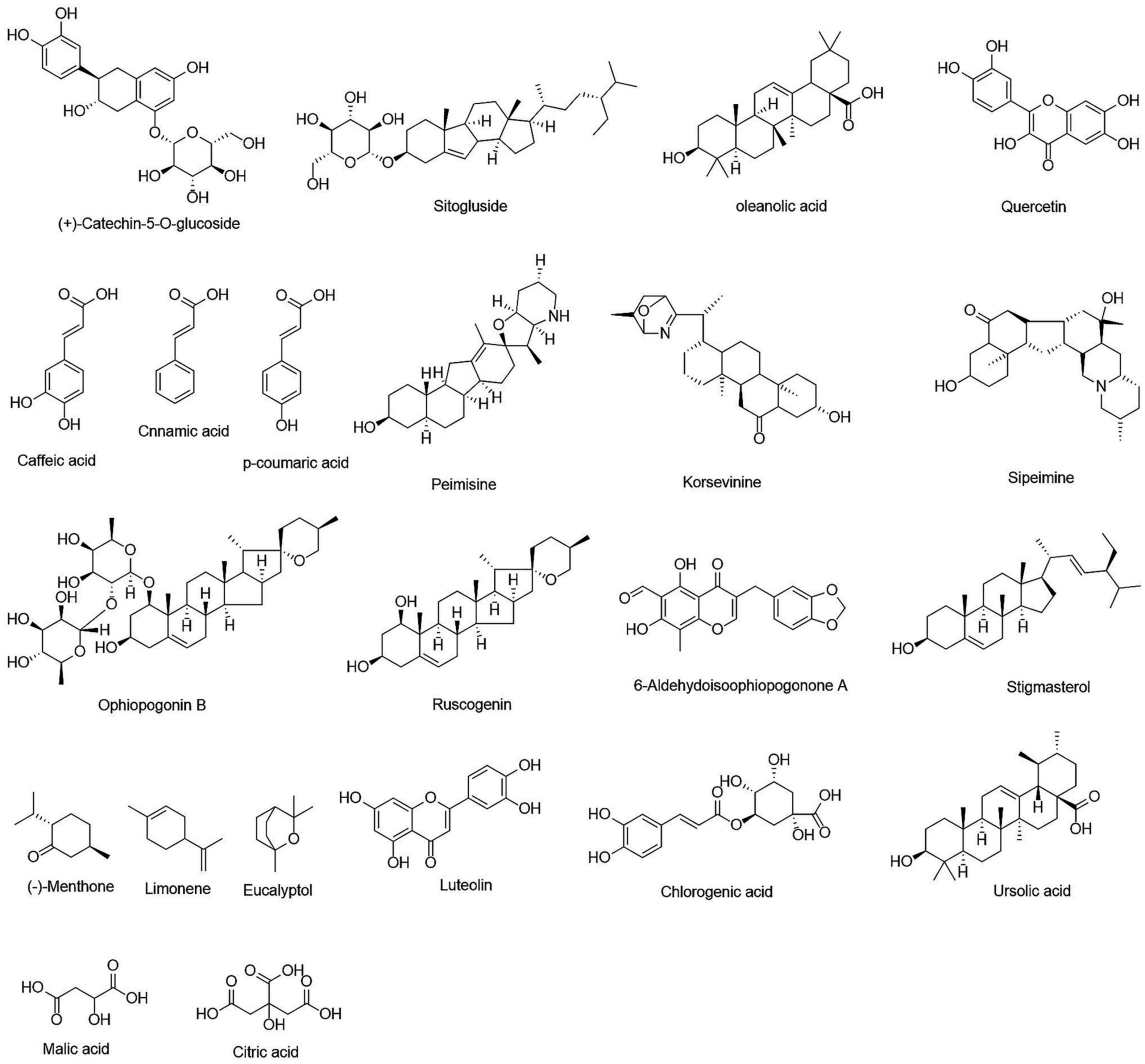
Screening and identification of potential active constituents in TSCP.

**Figure 4:**
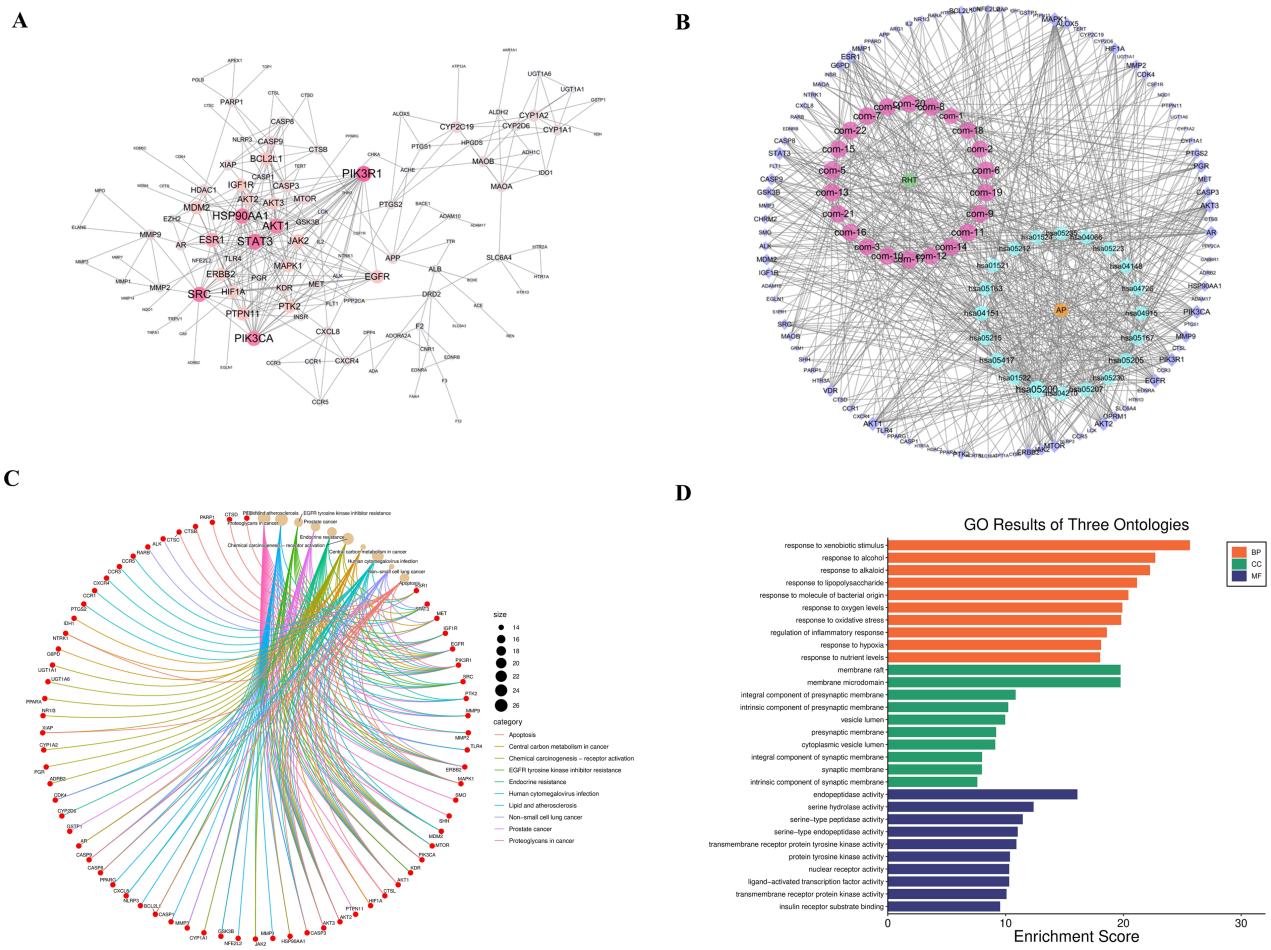
Identification of potential therapeutic targets for acute pharyngitis. **(A)** Network pharmacology-based screening of putative therapeutic targets. **(B)** Topological analysis of the PPI network for candidate targets. **(C)** Results of the KEGG pathway enrichment analysis. **(D)** Functional enrichment of candidate targets based on Gene Ontology.

The active chemical components in the herbal parts of TSCP were selected from the TCMSP database based on OB and DL; ultimately, 22 active ingredients were identified (Figure 3). The detailed sources of these 22 compounds are as follows: (+)-catechin-5-O-glucoside is derived from jujube (*Ziziphus jujuba*); Sitogluside is derived from *Mentha haplocalyx*, *Fritillaria taipaiensis*, jujube, persimmon sugar, and *Ophiopogon japonicus*; oleanolic acid is derived from *Mentha haplocalyx*, jujube, and persimmon sugar; Quercetin is derived from crispy pear (*Pyrus bretschneideri*), lotus root (*Nelumbo nucifera*), jujube, and *Mentha haplocalyx*; Caffeic acid is derived from *Mentha haplocalyx*, lotus root, jujube, persimmon sugar, and *Fritillaria taipaiensis*; Cinnamic acid is derived from *Mentha haplocalyx*, lotus root, and jujube; p-coumaric acid is derived from lotus root, jujube, and persimmon sugar; Peimisine, Korsevinine, and Sipeimine are all derived from *Fritillaria taipaiensis*; Ophiopogonin B, Ruscogenin, and 6-Aldehydoisoophiopogonone A are all derived from *Ophiopogon japonicus*; Stigmasterol is derived from *Ophiopogon japonicus*, *Fritillaria taipaiensis*, and jujube; (−)-Menthone, Limonene, Eucalyptol, and Luteolin are all derived from *Mentha haplocalyx*; Chlorogenic acid is derived from *Mentha haplocalyx*, lotus root, and crispy pear; Ursolic acid is derived from jujube, persimmon sugar, and *Mentha haplocalyx*; Malic acid is derived from crispy pear, honey, and jujube; Citric acid is derived from crispy pear, honey, and jujube. As shown in Figure 4, a total of 2,115 disease-related targets were identified. By combining the “ingredient-target” mapping relationship set with the “disease-target” mapping relationship set, a total of 174 common target nodes were obtained. A protein–protein interaction (PPI) network was constructed, and after removing disconnected nodes, it comprised 174 nodes and 333 edges, with an average node degree of 3.83. The PPI enrichment showed that the *p*-value is less than 1.0e^−16^. Gene Ontology (GO) functional enrichment analysis (Figure 4D) showed that the biological processes (BP) mainly included response to xenobiotic stimulus, negative regulation of apoptotic process, response to hypoxia, and response to lipopolysaccharide. Cellular components (CC) were primarily enriched in the plasma membrane, dendrite, membrane raft, and neuronal cell body. The majority of molecular functions (MF) were associated with identical protein binding, peptidase activity, enzyme binding, and transmembrane receptor protein tyrosine kinase activity. The Kyoto Encyclopedia of Genes and Genomes (KEGG) pathway enrichment analysis (Figure 4C) showed that the predicted targets were significantly involved in core inflammatory signalling pathways such as PI3K-Akt and NF-κB, as well as pathways including pathways in cancer, proteoglycans in cancer, and lipid and atherosclerosis.

### 3.4 TSCP activated the PI3K-Akt pathway and suppressed the NF-κB pathway to inhibit expression of iNOS, COX-2, and IL-6

**Figure 5:**
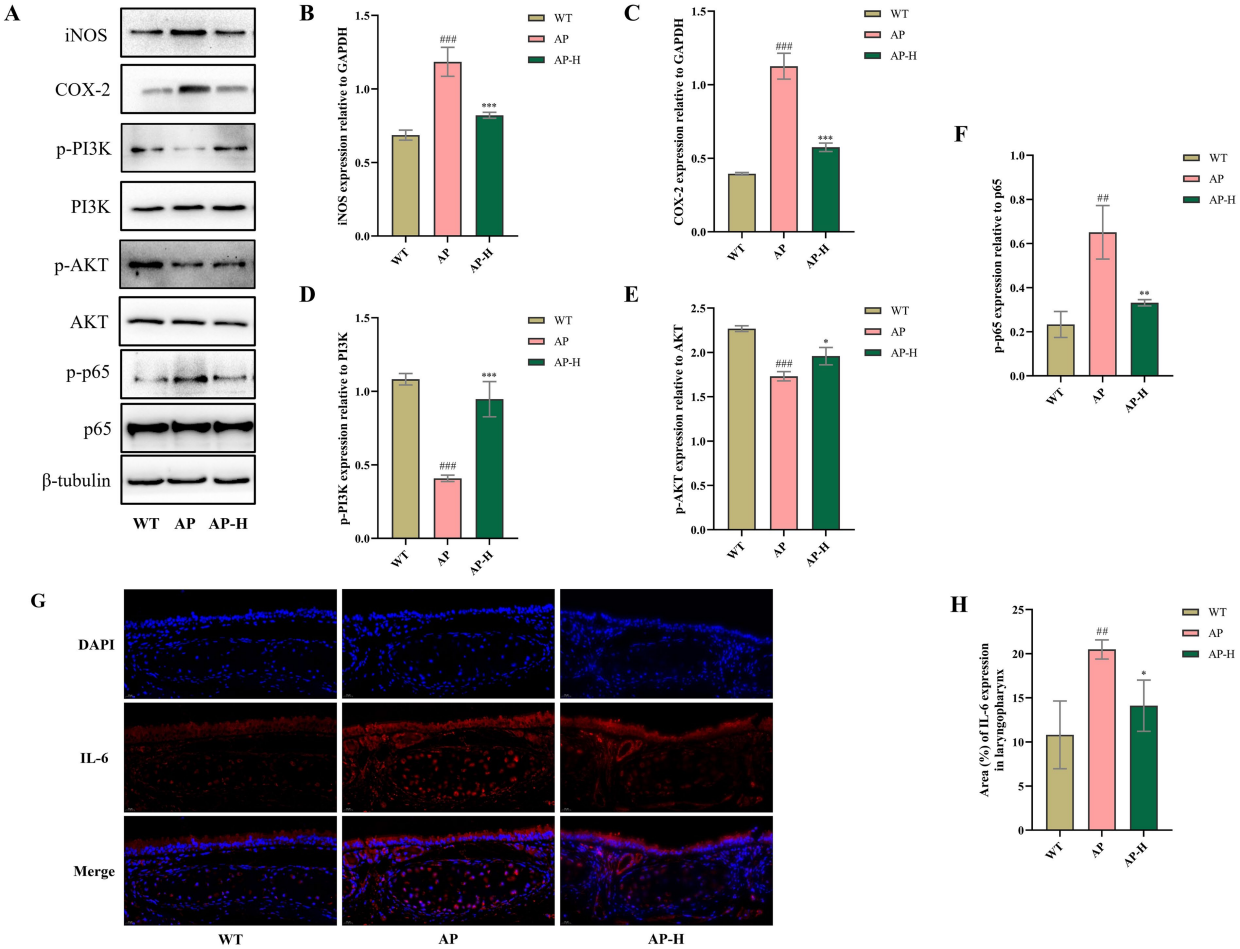
Impact of TSCP on the expression of inflammatory mediators and pivotal proteins in pharyngeal tissues from mice with pharyngitis. **(A)** Improvement in the expression of acute pharyngitis-associated proteins by TSCP. **(B)** iNOS expression relative to GAPDH. **(C)** COX-2 expression relative to GAPDH. **(D)** p-PI3K expression relative to PI3K. **(E)** p-AKT expression relative to AKT. **(F)** p-p65 expression relative to p65. **(G)** Representative immunofluorescence images showing IL-6 expression in pharyngeal tissue. **(H)** TSCP modulates IL-6 expression levels. Compared with the WT group, ^#^*p* < 0.05, ^##^*p* < 0.01, ^###^*p* < 0.001; compared with the AP model group ^*^p < 0.05, **p < 0.01, ^***^p < 0.001. *p* > 0.05 indicates no significant difference, and such comparisons are not marked.

Based on previous behavioural and histopathological observations, as well as the target prediction results mentioned above, we chose the AP-H group for subsequent experimental validation of the key targets. As shown in Figure 5G, immunofluorescence detection of IL-6 showed that the AP model group had a substantial increase in fluorescence intensity and protein expression in the pharyngeal tissue compared to the WT group (*p* < 0.01). AP-H group treatment significantly reduced IL-6 expression compared to the AP model group (*p* < 0.05). The above results show that TSCP significantly lowers the level of IL-6 in the pharyngeal tissue of mice with acute pharyngitis (Figure 5H). To further verify the core targets identified by network pharmacology, related pathways were investigated. As shown in Figure 5, compared with the WT group, the AP model group showed significantly decreased protein levels of the ratios of p-PI3K to total PI3K and p-AKT to total AKT, while simultaneously exhibiting significantly increased expression of p-p65 (*p* < 0.01), iNOS, and COX-2 (all *p* < 0.001). Compared with the AP model group, the AP-H group had a marked increase in p-PI3K/PI3K and p-AKT/AKT expression (both *p* < 0.001), while p-p65 (*p* < 0.05), iNOS, and COX-2 (all *p* < 0.001) were significantly reduced.

### 3.5 TSCP modulated inflammatory blood cell profiles in mice with acute pharyngitis

**Figure 6:**
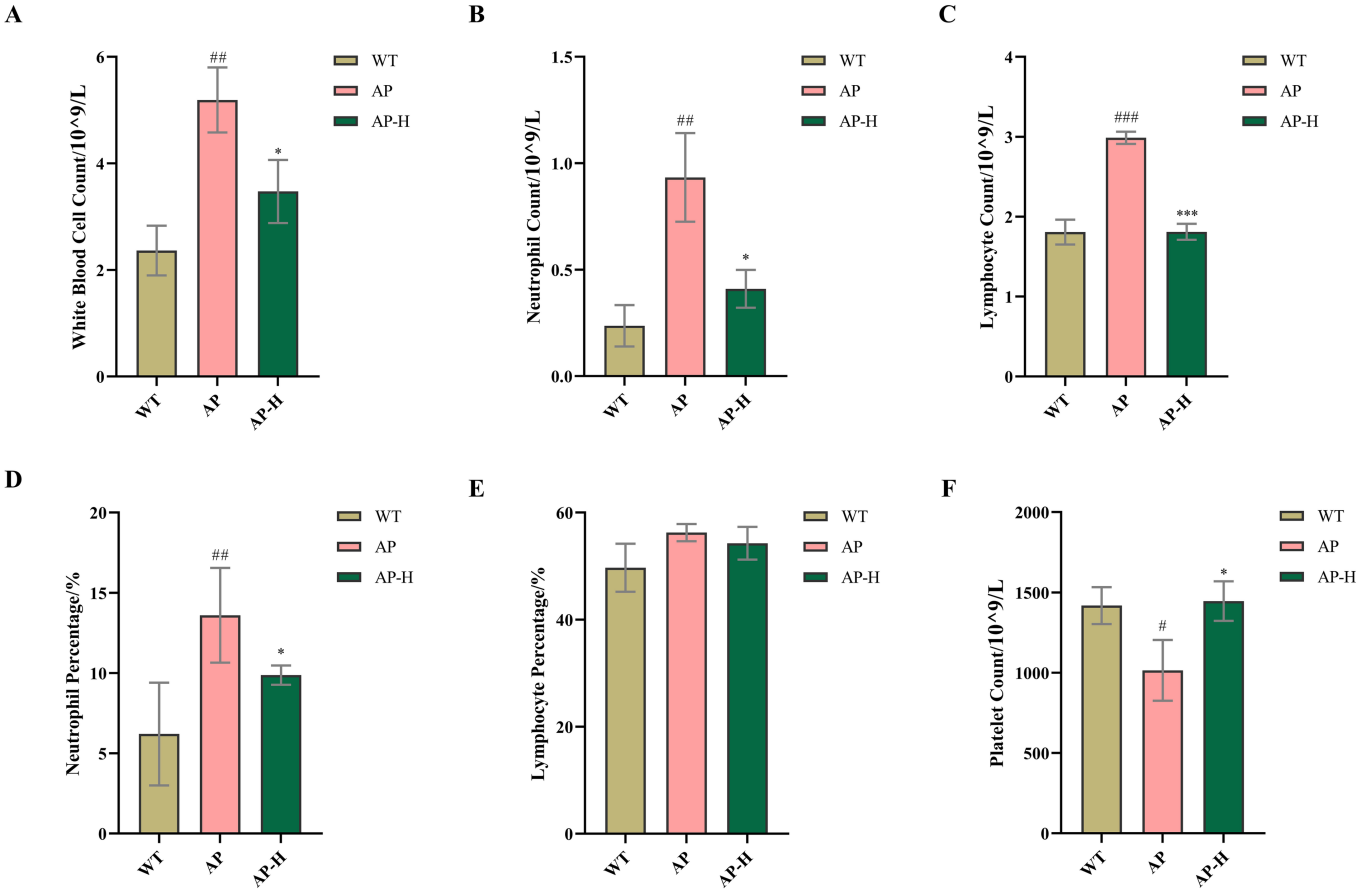
Results of blood cell analysis. **(A)** White blood cell count. **(B)** Neutrophil count. **(C)** Lymphocyte count. **(D)** Neutrophil percentage. **(E)** Lymphocyte percentage. **(F)** Platelet count. Compared with the WT group, ^#^*p* < 0.05, ##*p* < 0.01, ^###^*p* < 0.001; compared with the AP model group ^*^p < 0.05, **p < 0.01, ^***^p < 0.001. *p* > 0.05 indicates no significant difference, and such comparisons are not marked.

As shown in Figure 6, complete blood count analysis (detailed in Supplementary Table S1) showed that, compared with the WT group, the AP model group had significantly higher levels of white blood cells (WBCs), lymphocytes, and neutrophils, as well as a higher proportion of neutrophils (all *p* < 0.01). The platelet count was significantly reduced (*p* < 0.05). Although the absolute lymphocyte count increased significantly, the lymphocyte percentage did not change, likely because the total white blood cell count increased proportionally. Compared with the AP model group, the AP-H group had significantly lower WBC (*p* < 0.05), lymphocyte (*p* < 0.001), and neutrophil counts (*p* < 0.05), as well as a significantly decreased neutrophil percentage (*p* < 0.05). There was a substantial increase in the platelet count post-treatment (*p* < 0.05), but no variation was observed for lymphocytes. High-concentration TSCP treatment corrected the inflammatory cell abnormalities in the blood of mice with acute pharyngitis to a normal state.

### 3.6 The *in vitro* antibacterial activity of TSCP

The antibacterial activity of TSCP was evaluated against *S. aureus* MRSA and *E. coli* using MIC and MBC assays, biofilm inhibition tests, and confocal microscopy.

**Figure 7:**
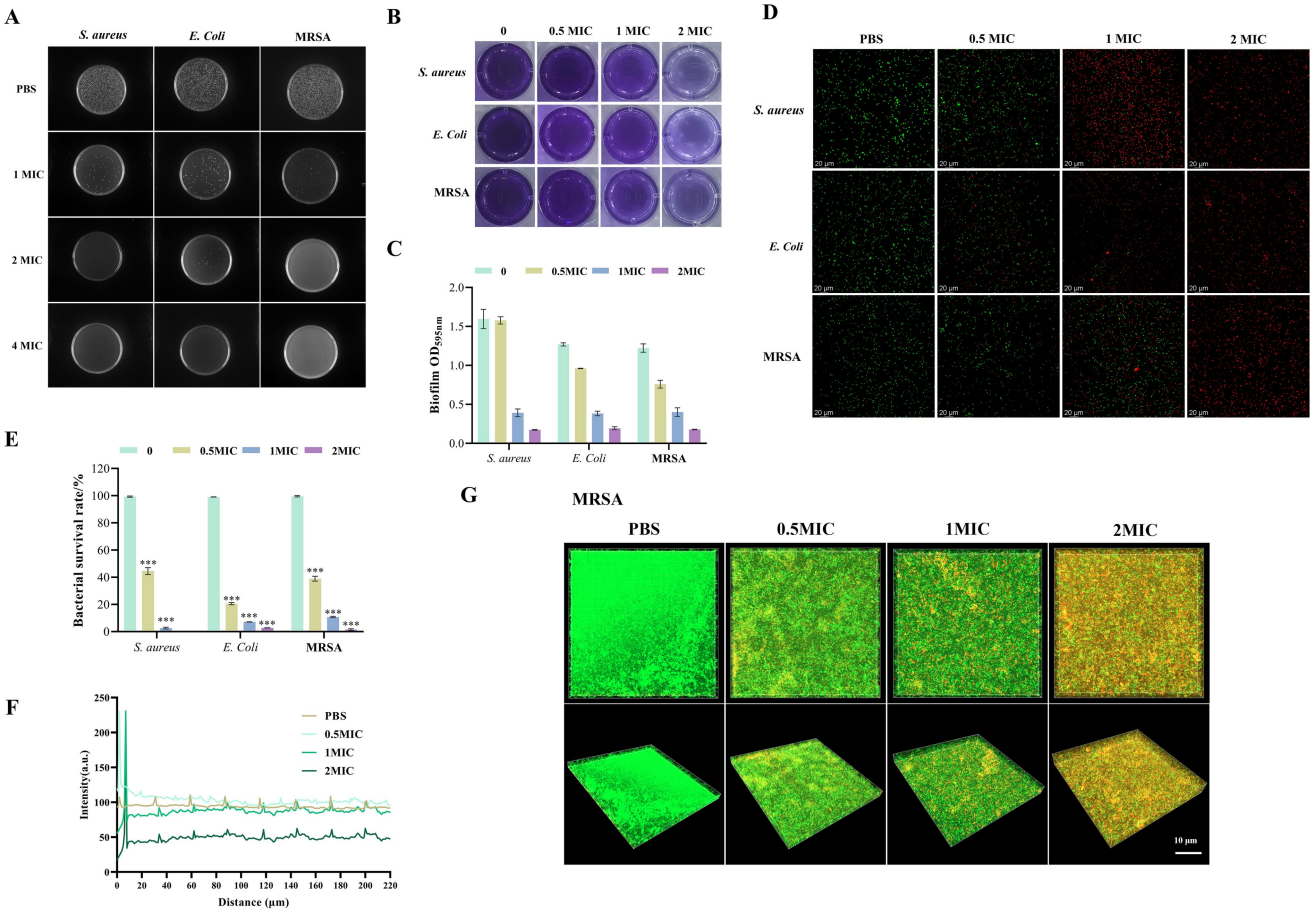
Antibacterial and anti-biofilm activity of TSCP against *S. aureus*, MRSA and *E. coli*. **(A)** Colony growth on agar plates after treatment with different concentrations of TSCP. **(B)** Representative wells showing biofilm formation by crystal violet staining. **(C)** Quantitative analysis of biofilm biomass (OD₅₉₅). **(D)** Fluorescence staining of live (green) and dead (red) bacteria. **(E)** Bacterial survival rate. **(F)** Bacterial fluorescence intensity as a function of scanning distance. **(G)** MRSA biofilm. Compared with the PBS group *p < 0.05, **p < 0.01, ***p < 0.001. *p* > 0.05 indicates no significant difference, and such comparisons are not marked.

As summarized in Table 1, TSCP exhibited concentration dependent antibacterial activity. The MIC values for *S. aureus*, MRSA and *E. coli* were 32, 64 and 256 μg/mL, respectively. The MBC for *S. aureus* was 64 μg/mL (2 × MIC), for MRSA was 128 μg/mL (2 × MIC), and for *E. coli* was 1,024 μg/mL (4 × MIC). The MBC/MIC ratios (2 for staphylococci and 4 for *E. coli*) indicate that TSCP exerts bactericidal activity against all three strains. The MBC was further confirmed by agar plate subculture. For *S. aureus* and MRSA, no colonies were present on plates from treatments at 1 × MIC and above, confirming the MBC values (2 × MIC relative to the MIC in Table 1). For *E. coli*, colonies were still visible at 2 × MIC but completely absent at 4 × MIC (Figure 7A), consistent with an MBC of 4 × MIC.

**Table 1:** MIC and MBC of TSCP against tested bacteria.

Bacterial strain	MIC (μg/mL)	MBC (μg/mL)	MBC/MIC
*S. aureus* (ATCC12600)	32	64	2
MRSA (ATCC43300)	64	128	2
*E. coli* (CVCC216)	256	1,024	4

As shown in Figures 7B,C, the effect of TSCP on biofilm formation was quantified by crystal violet staining and the absorbance was measured at 595 nm. The OD₅₉₅ values for each strain at different concentrations are summarized in Figure 7C. At 0.5 × MIC, biofilm biomass decreased by approximately 17.5, 24.5 and 37% for *S. aureus*, *E. coli* and MRSA, respectively, compared with the control. At 1 × MIC, the inhibition rate exceeded 60% for all strains, and at 2 × MIC it reached >80%, with MRSA biofilm biomass reduced to less than 15% of the control level. These findings indicate that TSCP effectively suppresses biofilm formation, potentially reducing bacterial colonization and resistance.

Fluorescence microscopy revealed that TSCP induced dose-dependent bacterial death. The PBS control group showed predominantly green fluorescence (live bacteria), indicating high viability. At 0.5 × MIC, the proportion of red fluorescence increased markedly, with a death rate exceeding 40%. At 2 × MIC, almost all bacteria displayed red fluorescence, achieving nearly 100% killing, which is consistent with the MBC value (2 × MIC). These results confirm that TSCP is bactericidal at the MBC concentration (Figures 7D,E).

Given the strong association between MRSA and recurrent acute pharyngitis, the effect of TSCP on MRSA biofilm architecture and membrane integrity was further examined by CLSM. In the control group, a dense and uniform biofilm with predominantly green fluorescence (live bacteria) was observed. After TSCP treatment, green fluorescence gradually decreased, while red/yellow fluorescence (membrane-damaged/dead bacteria) increased, accompanied by progressive disruption of the biofilm structure. At 2 × MIC (MBC), the biofilm was severely disrupted, with live bacterial fluorescence intensity reduced to less than 10% of the control level. These results demonstrate that TSCP kills MRSA by disrupting cell membrane integrity (Figures 7F,G).

## 4 Discussion

Considering that pharyngitis is widespread worldwide, and there are certain deficiencies in current conventional therapy, such as drug resistance, side effects, and frequent recurrence, this study was carried out to develop the TSCP. TSCP is derived from the optimised pear paste formula in the “Collection of Qing Palace Formulas”, mainly composed of medicinal and edible natural ingredients, and safety is given top priority. Using an established ammonia-induced murine model of acute pharyngitis, it was shown that TSCP reduced disease-related behavioural alterations and histopathological injury in a dose-dependent manner. To systematically explain the mechanism of action, research subsequently focused on the high-concentration treatment group. Combining network pharmacology prediction with molecular biology verification and *in vitro* microbiological experiments provided a multidisciplinary study that systematically elucidates the anti-pharyngitis activity of TSCP at both the level of its *in vivo* therapeutic effects and direct bactericidal action.

Based on previous research that has shown that extracts from *Hosta plantaginea* flowers can alleviate pharyngeal inflammation by regulating NF-κB and MAPKs and by inhibiting the PI3K-Akt pathway (Wang J. et al., 2024), and reports that peimine can reduce respiratory inflammatory injury by regulating NF-κB and PI3K-AKT signaling and interacting with COX-2 and iNOS proteins (Xiang et al., 2022; Feng et al., 2025), and also considering that the active components of *Ophiopogon japonicus* (such as total saponins) have been found to inhibit the PI3K/AKT/mTOR axis, thereby alleviating radiation-induced pulmonary inflammatory cell infiltration and fibrosis (Chen et al., 2017; Yao et al., 2019), we further explored the mechanism of TSCP. In view of this, network pharmacology was used to screen out 22 potential active ingredients from the formula and determine that there are 174 key targets, constructing a three-dimensional regulatory network of “active components-targets-signaling pathways-acute pharyngitis”. Molecular biology experiments confirmed that TSCP can activate the PI3K/AKT pathway, then regulate NF-κB activity and inhibit the expression of downstream inflammatory proteins such as iNOS, COX-2, and IL-6. While these data demonstrate that TSCP modulates the PI3K/AKT and NF-κB pathways, further validation using specific inhibitors or activators is required to establish causality. In addition, future studies will include a positive control group (e.g., a standard anti-inflammatory drug) to further validate the mechanism of action. These experiments are planned as part of our ongoing mechanistic investigation of TSCP. However, the antibacterial mechanism of TSCP, particularly the specific active components responsible for disrupting bacterial membrane integrity and biofilm formation, remains to be further identified. Additionally, the *in vitro* antibacterial efficacy has not been validated in a bacterial pharyngitis animal model, which limits the direct translation of these findings to clinical application.

At the same time, the observed reduction in circulating white blood cells, lymphocytes, and neutrophils in the TSCP treatment group could be explained in two ways. It might be a direct immunomodulatory effect of absorbed TSCP metabolites, or it could simply be a secondary consequence of resolving local pharyngeal inflammation. Due to the ammonia-induced model produces a strong local inflammatory response, we lean toward the latter—the drop in systemic leukocytes probably reflects reduced inflammatory cytokine release from the pharyngeal mucosa, rather than a direct action on bone marrow or peripheral immune cells. In future, the pharmacokinetic studies will be needed to clarify whether TSCP components actually enter the circulation and directly modulate immune cells. Additionally, the increased platelet count after TSCP administration may have biological significance beyond hemostasis, as platelets release growth factors and cytokines that promote tissue repair and angiogenesis (Burnouf et al., 2023). Thus, elevated platelet levels could contribute to the mucosal healing observed in TSCP-treated mice, the underlying mechanisms require further investigation.

Respiratory pathogens including *Staphylococcus aureus*, methicillin-resistant *S. aureus* (MRSA) and *Escherichia coli* are commonly isolated from patients with acute and recurrent pharyngitis, which are closely associated with inflammatory progression, persistent mucosal colonization and disease delayed recovery. Among them, *Staphylococcus aureus* is a major pathogen in pharyngitis, contributing to the onset and recurrence of acute infections through robust biofilm formation and high-level antibiotic resistance (Wang Z. et al., 2024; Brdová et al., 2024; Al-Sammarraie and Al-Nuaimi, 2023). In the present study, we further expanded the antimicrobial spectrum of TSCP by introducing methicillin-resistant *S. aureus* (MRSA ATCC 43300) and *Escherichia coli* (CVCC 216) alongside standard *S. aureus* ATCC 12600. The MIC values of TSCP against *S. aureus*, MRSA and *E. coli* were determined to be 32, 64 and 256 μg/mL, respectively, with corresponding MBC values twice the MIC for each strain, indicating a clear concentration-dependent bactericidal transition characteristic. Consistent with the MIC/MBC results, TSCP exhibited more potent inhibitory and bactericidal effects against Gram-positive strains including MRSA than Gram-negative *E. coli*, which may be attributed to the outer membrane barrier of Gram-negative bacteria limiting the penetration of active phytochemicals. *In vitro* functional assays further verified that TSCP inhibited bacterial growth, damaged membrane integrity, reduced bacterial adhesion, and severely disrupted biofilm microstructure in a concentration-dependent manner across all three tested strains. These broad-spectrum antibacterial and anti-biofilm activities, especially against drug-resistant MRSA, are driven by the synergistic interaction of multiple active components within TSCP, which may help block pathogen colonization, alleviate persistent pharyngeal infection, and offer a promising plant-based alternative strategy to combat antibiotic-resistant pathogens associated with recurrent pharyngitis.

In summary, using a murine model of acute pharyngitis, this study demonstrates that TSCP exerts potent anti-inflammatory effects by modulating the PI3K-AKT/NF-κB signaling pathway. Hematological analysis and histopathological examination confirmed its ability to promote mucosal repair. Additionally, TSCP exhibits notable antibacterial activity, reducing bacterial adhesion and disrupting biofilm structure. These findings provide experimental evidence supporting the modern application of traditional herbal medicine. Given its multi-target mechanism of action, TSCP may offer a new approach for treating drug-resistant *Staphylococcus aureus*-associated acute pharyngitis. It should be noted that the ammonia-induced pharyngitis model used herein does not fully recapitulate bacterial infection. Therefore, future studies employing an *S. aureus*-induced bacterial pharyngitis model are warranted to determine whether the antibacterial and anti-biofilm activities observed “*in vitro*” translate into “*in vivo*” efficacy. Furthermore, long-term interventions in chronic pharyngitis models within the framework of “modernization of Qin Medicine” will be conducted to expand the clinical applicability of TSCP and improve therapeutic strategies for current diseases.

## Data Availability

The original contributions presented in the study are included in the article/Supplementary material, further inquiries can be directed to the corresponding authors.

## Footnotes

1
https://old.tcmsp-e.com/tcmsp.php
