# Supplementary material for: A traditional botanical formula attenuates acute pharyngitis in mice through anti-inflammatory and anti-staphylococcal activities
Source: Front Microbiol. 2026 May 22;17:1841770. doi: 10.3389/fmicb.2026.1841770 (PMC13236933; doi:10.3389/fmicb.2026.1841770)
Supplement: Supplementary file 1 [file Data_Sheet_1.docx]

**A traditional botanical formula attenuates acute pharyngitis in mice through anti-inflammatory and anti-staphylococcal activities**

**Xinyu Zhang^1,2^, Wenyuan Li^1,3^, Qinghua Xie^1,3^, Yifei Zhang****^1,4^, Zerun Wang^1,5^, Wan Wang^1,4^, Le Wang^1,4^, Ziqi Zhang^1^*, Rui Han^1^***

^1.Shaanxi Key Laboratory of Brain Disorders & Institute of Basic and Translational Medicine, Xi’an Medical University, Xi’an 710021, China^

^2.School of Stomatology, Xi’an Medical University, Xi’an 710021, China^

^3.School of General Medicine, Xi’an Medical University, Xi’an 710021, China^

^4.School of Pharmacy, Xi’an Medical University, Xi’an 710021, China^

^5.School of Clinical Medicine, Shandong Second Medical University, Weifang 261000, China^

^Xinyu Zhang and Wenyuan Li contributed equally to this work.^

**^*Correspondence：^**^Ziqi Zhang, E-mail: zhangziqi@xiyi.edu.cn；Rui Han, E-mail: hanrui@xiyi.edu.cn; hruigsyc@163.com.^

The counting result of complete blood cell count performed on mice via abdominal blood collection. In the blood cell analysis results, each type of blood cell unit is expressed as 10^9/L. In the WT group, the total number of white blood cells was 2.15±1.25, the total number of lymphocytes was 1.96±0.15, the total number of Neutrophils was 0.13±0.19, the total number of platelets was 1084±15; In the AP model group, the total number of white blood cells was 5.19±0.61, the total number of lymphocytes was 2.92±0.15, the total number of Neutrophils was 0.70±0.30, the total number of platelets was 1418±237; In the AP-H model group, the total number of white blood cells was 3.14±0.13, the total number of lymphocytes was 1.81±0.10, the total number of Neutrophils was 0.31±0.13, the total number of platelets was 1547.50±122.50.

**Table S1 Hematological Parameters in Each Group of Mice (n=5)**

| Group | abbreviation | WT group | AP model group | AP-H group | Unit |
| --- | --- | --- | --- | --- | --- |
| White blood cell count | WBC | 2.15±1.25 | 5.19±0.61 | 3.14±0.13 | 10^9/L |
| Neutrophil count | Neu# | 0.13±0.19 | 0.70±0.30 | 0.31±0.13 | 10^9/L |
| Lymphocyte count | Lym# | 1.96±0.15 | 2.92±0.15 | 1.81±0.10 | 10^9/L |
| Monocyte count | Mon# | 0.82±0.79 | 1.66±0.36 | 0.61±0.22 | 10^9/L |
| Eosinophil count | Eos# | 0.36±0.12 | 0.51±0.06 | 0.31±0.03 | 10^9/L |
| Basophil count | Bas# | 0.01 | 0.00 | 0.01±0.01 | 10^9/L |
| Neutrophil percentage | Neu% | 6.20±3.20 | 13.6±2.95 | 9.87±0.60 | % |
| Lymphocyte percentage | Lym% | 49.70±4.50 | 56.26±1.60 | 57.60±6.00 | % |
| Monocyte percentage | Mon% | 18±6.5 | 6.70±5.00 | 19.75±6.55 | % |
| Eosinophil percentage | Eos% | 10.65±0.15 | 9.90±0.75 | 10.05±0.45 | % |
| Basophil percentage | Bas% | 0.45±0.05 | 0.0 | 0.50±0.40 | % |
| Red blood cell count | RBC | 7.18±0.64 | 7.29±0.42 | 7.32±0.07 | 10^12/L |
| hemoglobin | HGB | 117±12 | 118±4 | 109.50±4.50 | g/L |
| Hematocrit | HCT | 40.70±0.5 | 40.30±0.10 | 38.90±0.31 | % |
| Mean Corpuscular Volume | MCV | 57.10±4.40 | 55.2±2.95 | 53.20±0.10 | fL |
| Mean Corpuscular Hemoglobin | MCH | 16.20±0.20 | 16.20±0.30 | 15.00±0.50 | pg |
| Mean Corpuscular Hemoglobin Concentration | MCHC | 286±26 | 296±11 | 282±10 | g/L |
| Red Blood Cell Distribution Width - Coefficient of Variation | RDW-CV | 22.20±4.5 | 16.40 | 21.10±2.10 | % |
| Red Blood Cell Distribution Width - Standard Deviation | RDW-SD | 52.55±14.45 | 37.45±1.95 | 46.15±4.85 | fL |
| Platelet count | PLT | 1084±15 | 1418±237 | 1547.50±122.50 | 10^9/L |
| Mean Platelet Volume | MPV | 8.70±1.10 | 6.90±1.61 | 7.30±0.20 | fL |
| Platelet Distribution Width | PDW | 16.20±0.10 | 15.50±0.60 | 15.55±0.15 |  |
| Plateletcrit | PCT | .*** | 0.986±0.06 | .*** | % |


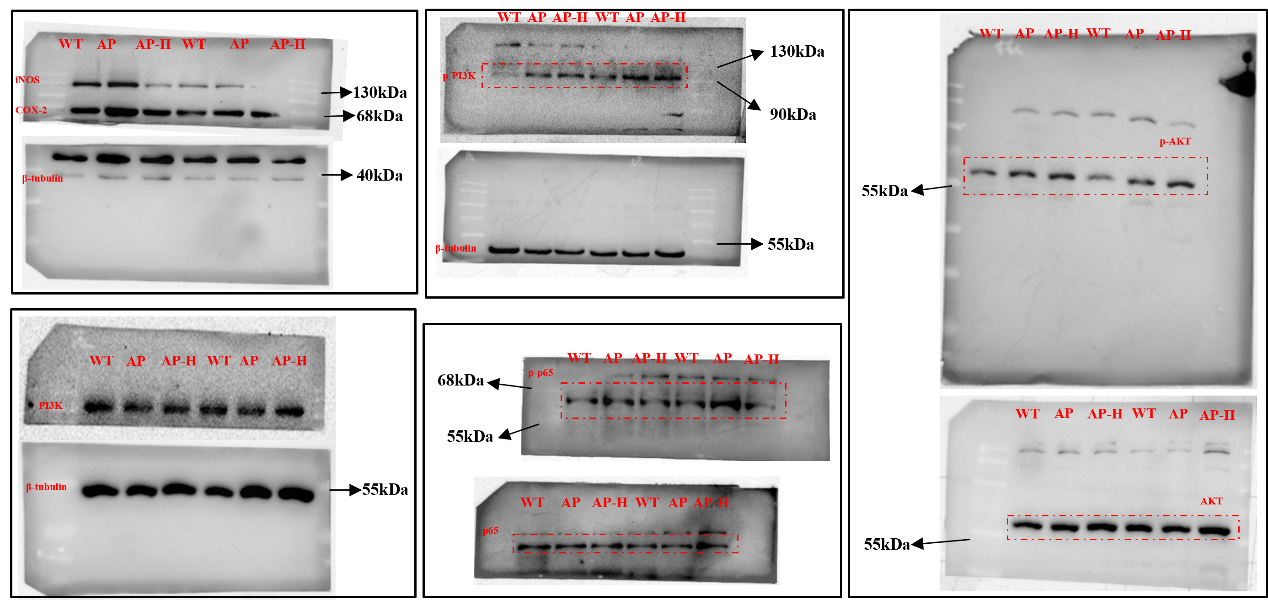


**Figure S1 The original images of Westren Blots for Figure 5A.**

To ensure the reliability of the results, samples were loaded in duplicate (technical replicates) on the same gel. The dashed box indicates the area that was cropped and presented in the main text (Figure 5A). All exposures and replicates are shown here to demonstrate the complete dataset.
